# Expression And Prognostic Role of PRDX1 In Gastrointestinal Cancers

**DOI:** 10.7150/jca.86568

**Published:** 2023-09-11

**Authors:** Zhou Zhang, Pengli Zhou, Mingyue Liu, Bing Pei

**Affiliations:** 1 Department of Clinical Laboratory, Wuxi Huishan District People's Hospital, Wuxi, Jiangsu Province, 214000, China.; 2 College of Basic Medicine, China Medical University, Shenyang, Liaoning province 110000, China.; 3 Department of Ultrasound, Wuxi No.2 People's Hospital; Jiangnan University Medical Center, Affiliated Wuxi Clinical College of Nantong University, Wuxi, Jiangsu province 214002, China.; 4 Department of Clinical Laboratory, The Affiliated Suqian First People's Hospital of Nanjing Medical University, Suqian, Jiangsu, 223800, China.

**Keywords:** PRDX1, gastrointestinal cancers, expression, prognosis.

## Abstract

Esophageal, gastric, liver, and colorectal cancers represent four prevalent gastrointestinal cancers that pose substantial threats to global health due to their high morbidity and mortality rates. Peroxiredoxin 1 (PRDX1), a significant component of the PRDXs family, primarily functions to counteract the peroxides produced by metabolic activities in the body, thereby maintaining the dynamic equilibrium of peroxides *in vivo*. Intriguingly, PRDX1 expression correlates strongly with cancer's onset, progression, and prognosis. This study mainly applied bioinformatics methods to analyze PRDX1's expression, diagnosis, and prognosis in gastrointestinal cancers and to summarize current research advancements. Evidence from the bioinformatics database suggested that the high expression of PRDX1 was a prominent characteristic of these four gastrointestinal cancers, with this observation reaching statistical significance. The high expression of PRDX1 in gastrointestinal cancer cells also confirms this result. Notably, the primary alteration in PRDX1 within these cancers is the presence of genetic mutations. PRDX1 demonstrated the highest diagnostic efficacy for colorectal cancer. Nevertheless, elevated PRDX1 levels only significantly diminished the survival time of liver cancer patients, exerting no statistically significant impact on the survival duration of patients afflicted by the other three types of gastrointestinal cancers. Recent research has indicated variability in PRDX1 expression across different cancer types, with high expression being predominantly observed in these four gastrointestinal cancers and, in most instances, unfavorable prognosis. These findings broadly align with the results derived from bioinformatics. This research underscores the high expression of PRDX1 in gastrointestinal cancers, its relevance to the diagnosis and prognosis monitoring of these cancers, and its potential to guide clinical treatment for these cancers.

## Introduction

Gastrointestinal cancers make up approximately 26% of all cancer incidences and contribute to about 35% of all cancer mortalities, a characteristic duality described as "double high"^1^. This group includes prevalent malignancies such as liver, gastric, esophageal, and colorectal cancer. Notwithstanding significant advances in colorectal cancer screening, the diagnosis of other gastrointestinal cancers, including liver, gastric, and esophageal cancer, often occurs at advanced stages, with patients experiencing a subsequent poor prognosis^2^, ^3^. Factors such as population growth, aging, and lifestyle changes have led to a continual rise in gastrointestinal cancer incidences, with an accompanying shift toward younger patient demographics^4^.

Reactive oxygen species (ROS) are primary oxidative stress products in the body and are widely recognized as critical contributors to cancer development and progression^5^ ^6^. The accumulation of ROS has a bifaceted impact on cells. A moderate ROS increase can stimulate cell proliferation and differentiation, while an excessive ROS concentration can lead to oxidative damage to DNA, proteins, and lipids^7^. The Peroxiredoxins (PRDXs) family, a group of common peroxidase enzymes, can modulate excessive ROS accumulation, thereby influencing the onset and progression of various diseases, including cancer. Therefore, targeting antioxidant mechanisms in cancer in combination with current cancer therapies may be a potential strategy to treat tumors^8^, ^9^. PRDX1, a member of the PRDXs family, resides at the 1q34.1 locus, weighs approximately 23 kDa, and is classified as a typical 2-Cys cysteine type. PRDX1 is a member of the 2-Cys PRDXs subfamily and is present mainly in the cytosol^10^. PRDX1 containing a cysteine at the N-terminal Cys52 detoxifies peroxides at the expense of Cys52 oxidation through intermolecular disulphide formation with the other conserved C-terminal Cys173 residue^11^.

Within the nucleus, oligomeric PRDX1 can either promote or inhibit cell death by directly binding transcription factors such as p53, c-Myc, NF-κB, and AR, thereby altering their biological activity. In the cytoplasm, PRDX1 in the form of dimers regulates ROS-dependent signaling pathways, thereby influencing cell growth, differentiation, proliferation, and apoptosis^11^. In the presence of reactive oxygen species (ROS) inducers, PRDX1 can promote PCa cell proliferation by increasing AR protein stability and activating AR signaling, even under androgen deprivation conditions^9^. Furthermore, PRDX1, via Toll-like receptors (TLR4), controls inflammation, immunity, and tissue repair processes, thereby augmenting the cytotoxic activity of NK cells against cancer cells^12^. Multiple studies have indicated that PRDX1 is overexpressed in several types of cancers including breast, prostate, lung, and liver cancers^9^, ^11^, ^13^-^16^. The level of its expression has a significant association with tumor cell proliferation, differentiation, metastasis, recurrence, and sensitivity to radiotherapy and chemotherapy^11^, ^17^-^19^. Despite these findings, a comprehensive review of PRDX1's role in common gastrointestinal cancers remains elusive.

Bioinformatics, a rapidly evolving discipline, applies methodologies and tools from human genome life science, information science, mathematics, and biology to acquire, process, store, analyze, and interpret vast quantities of biological data, thereby unraveling their biological significance^20^-^22^. Utilizing various biological databases, bioinformatics analysis can identify key tumor-related biomarkers, thereby establishing a foundation for cancer diagnosis and prognosis 23.

This study applies bioinformatics analysis to illuminate PRDX1 expression, diagnosis, and prognosis in gastrointestinal tumors. Subsequent analysis further scrutinizes the current research progression of PRDX1 in these malignancies, providing insights that may guide future exploration of PRDX1's role in gastrointestinal cancers.

## Materials and methods

### Expression and Mutation Analysis of PRDX1 in Pan-Cancer

The University of Alabama, Birmingham Cancer Data Analysis Portal website (UALCAN; http://ualcan.path.uab.edu/) was utilized to derive PRDX1 expression levels in pan-cancer and corresponding adjacent tissues. The cBioPortal website (https://www.cbioportal.org/) was subsequently used to acquire data on mutation frequency, mutation type, copy number alterations (CNA), and mutation location of PRDX1 in pan-cancer.

### Identification of Differentially Expressed Genes (DEGs) of PRDX1 in Gastrointestinal Cancers

Volcanic maps were created using The Cancer Genome Atlas (TCGA) databases to illustrate the distribution of differentially expressed genes in four types of gastrointestinal cancers. The expression of PRDX1 in gastrointestinal cancers was depicted using the TCGA databases, and a statistical difference analysis was conducted.

### Cell culture and reagents

TE-1 (Human esophageal cancer), HepG2 (human hepatocellular carcinoma) and LS513 (Human colorectal cancer) cell lines were purchased from National Collection of Authenticated Cell Cultures. HEEC (Human esophageal epithelium), NCM460 (Human colon epithelium), SGC-7901 (Human gastric cancer) and GES-1(Human gastric epithelium) cell lines were purchased from IMMOCELL. QSG7701 (Human hepatocyte) was purchased from Beyotime. Each cell line is cultured according to the conditions specified in their respective culture instructions.

### Reverse transcription-quantitative (RT-q) PCR

Total RNA was extracted from eight cell lines using TRIzol reagent (Beyotime, R0016). Using BeyoRT ™ The II cDNA first strand synthesis kit (Beyotime, D7168M) synthesizes complementary DNA (cDNA). BeyoFast ™ SYBR Green qPCR Mix (Beyotime, D7265) and CFX 96 thermal circulator (Bio Rad) were used for RT-qPCR. Perform RNA extraction, cDNA synthesis and qPCR according to the instructions. RT-qPCR was carried out using the following conditions: preheating for 2 min at 95°C; and then repeating 40 cycles in 95°C for 15 sec and 56°C for 30 sec. The primer sequences of PRDX1 were as follows: Forward, 5'-AGCTGTTATGCCAGATGGTC-3' and Reverse, 5'-TGAGAATCCACAGAAGCACC-3'. Using GAPDH as an internal reference gene and the primer sequence were as follows: Forward, 5'-ATTCCACCCATGGCAAATTC-3' and Reverse, 5'-TGGGATTTCCATTGATGACAAG-3'. The relative expression level of PRDX1 in this study was calculated using the 2^-ΔΔCq^ formula. Repeat the experiment three times.

### Gene Mutations and Related Genes Analysis of PRDX1 in Gastrointestinal Cancers

The cBioPortal website was initially employed to investigate the mutation sources and types of PRDX1 in gastrointestinal cancers. Subsequently, the UALCAN website showed the correlation and correlation coefficients of the primary related genes for PRDX1 in common gastrointestinal cancers.

### Clinical parameters of patients

We downloaded clinical data of 184 ESCA patients from TCGA, and then deleted 43 samples with incomplete clinical data, resulting in a valid sample of 141; We downloaded clinical data of 415 STAD patients from TCGA, and then deleted 70 samples with incomplete clinical data, resulting in a valid sample of 345; We downloaded clinical data of 371 LIHC patients from TCGA, and then deleted 141 samples with incomplete clinical data, resulting in a valid sample of 230; We downloaded clinical data of 622 COAD patients from TCGA, and then deleted 111 samples with incomplete clinical data, resulting in a valid sample of 511. The specific parameter characteristics are detailed in **Table 1.**

### Diagnostic Efficacy of PRDX1 in Gastrointestinal Cancers

The TCGA database was used to assess the diagnostic potential of PRDX1 in gastrointestinal cancers. This assessment was followed by plotting the Receiver Operating Characteristic (ROC) curve. The area under the curve (AUC) and the optimal cutoff value were specifically compared to determine the best diagnostic efficacy.

### Survival Analysis of PRDX1 in Gastrointestinal Cancers

The TCGA database was used to analyze the survival rates of patients with PRDX1 in gastrointestinal cancers.

### Literature Review

To further consolidate understanding of PRDX1's role in common gastrointestinal cancers, relevant literature was examined, focusing on esophageal cancer, gastric cancer, liver cancer, and colorectal cancer. This analysis aimed to summarize the research progress of PRDX1 in these common gastrointestinal cancers.

### Statistical Analysis

The R software (4.0.2) and Graphpad 9.0 were employed for data analysis to evaluate statistical differences. Data are expressed as mean ± standard deviation. The unpaired Student's t-test was used for data analysis. ROC curves were utilized to evaluate the diagnostic efficacy of PRDX1 in gastrointestinal cancers. The Kaplan-Meier plots were applied to illustrate the survival curve of PRDX1 in these cancers. All hypothesis tests were two-sided, with P<0.05 being considered indicative of a statistically significant difference. Gene differential analysis [|LogFC|>1, adjusted P-value (FDR) <0.05] was conducted by comparing tumor tissue with controls using the limma R package. The entire gene expression data underwent log2 transformation, with correlations calculated using Spearman's coefficient analysis.

## Result

### Expression and Mutation of PRDX1 in Pan-Cancer

**Figure 1:**
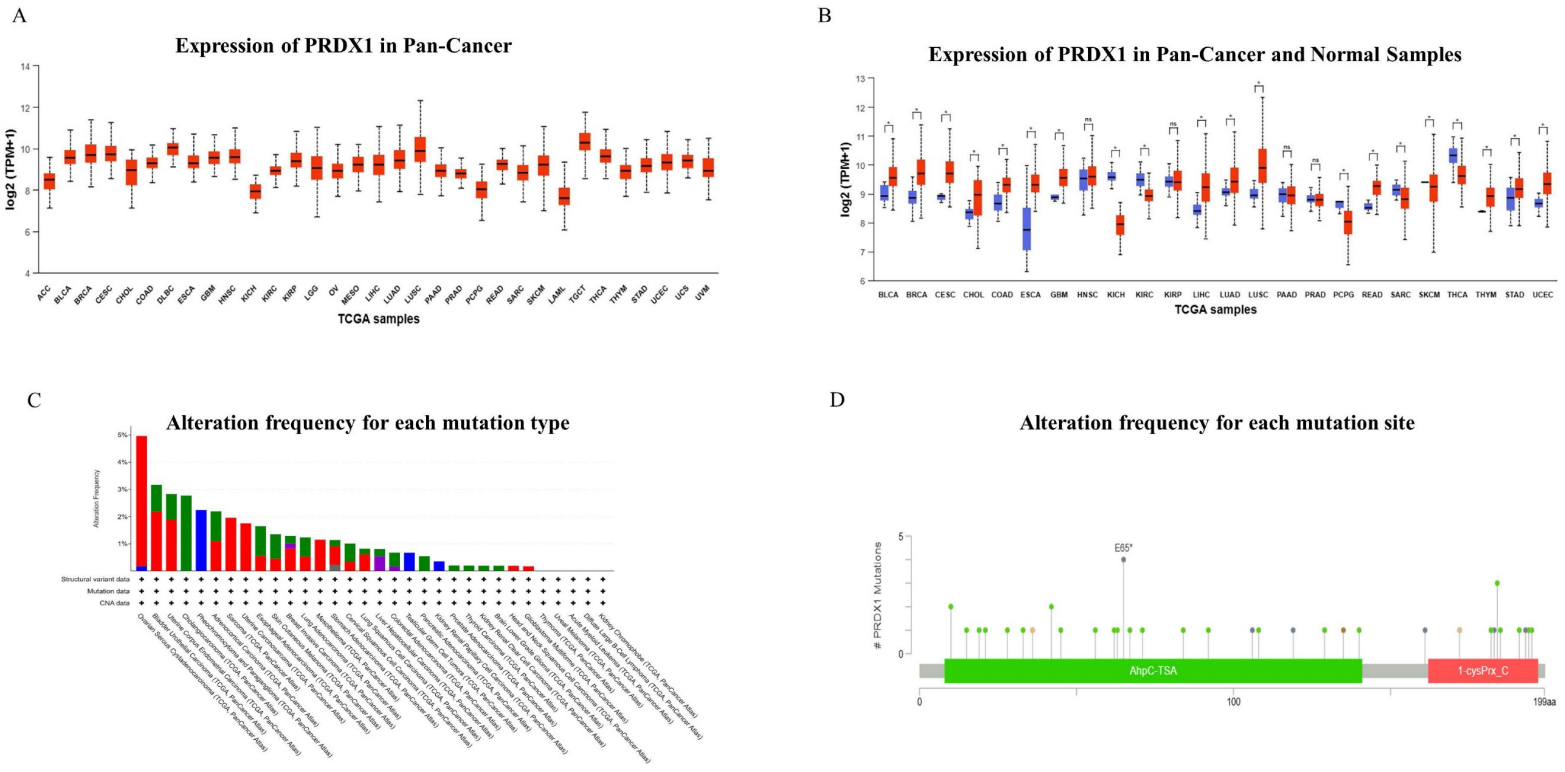
Expression and Mutation of PRDX1 in Pan-Cancer. **(A)** Expression of PRDX1 in Pan-Cancer. **(B)** Expression of PRDX1 in Pan-Cancer and Normal Samples. **(C)** The alteration frequency for each mutation type and **(D)** mutation site. *P<0.05; “ns” represents no statistical significance.

The expression of PRDX1 in pan- cancer was detected, and the results showed that the expression of PRDX1 in gastrointestinal cancers was higher than the median level of pan-cancer (**Figure 1A**). When incorporating adjacent tissues for comparison, it was revealed that the expression of PRDX1 in pan-cancer typically exceeds that in corresponding adjacent tissues. Notably in gastrointestinal cancers, PRDX1 expression surpasses that in corresponding adjacent tissues (**Figure 1B**). The mutation frequency, mutation type, and copy number alterations (CNA) of PRDX1 in pan-cancer were also investigated. Findings highlighted that PRDX1 is primarily characterized by gene mutations in gastrointestinal cancers (46.67% gene mutations, 26.67% gene amplification, 20% structural variant, and 6.67% multiple alterations) (**Figure 1C**). Further analysis of mutation location based on PRDX1's structure revealed that the E65* alteration in the AhpC-TSA domain is the primary type of genetic alteration of PRDX1 (**Figure 1D**).

### Identification of Differentially Expressed Genes (DEGs) of PRDX1 in Gastrointestinal Cancers

**Figure 2:**
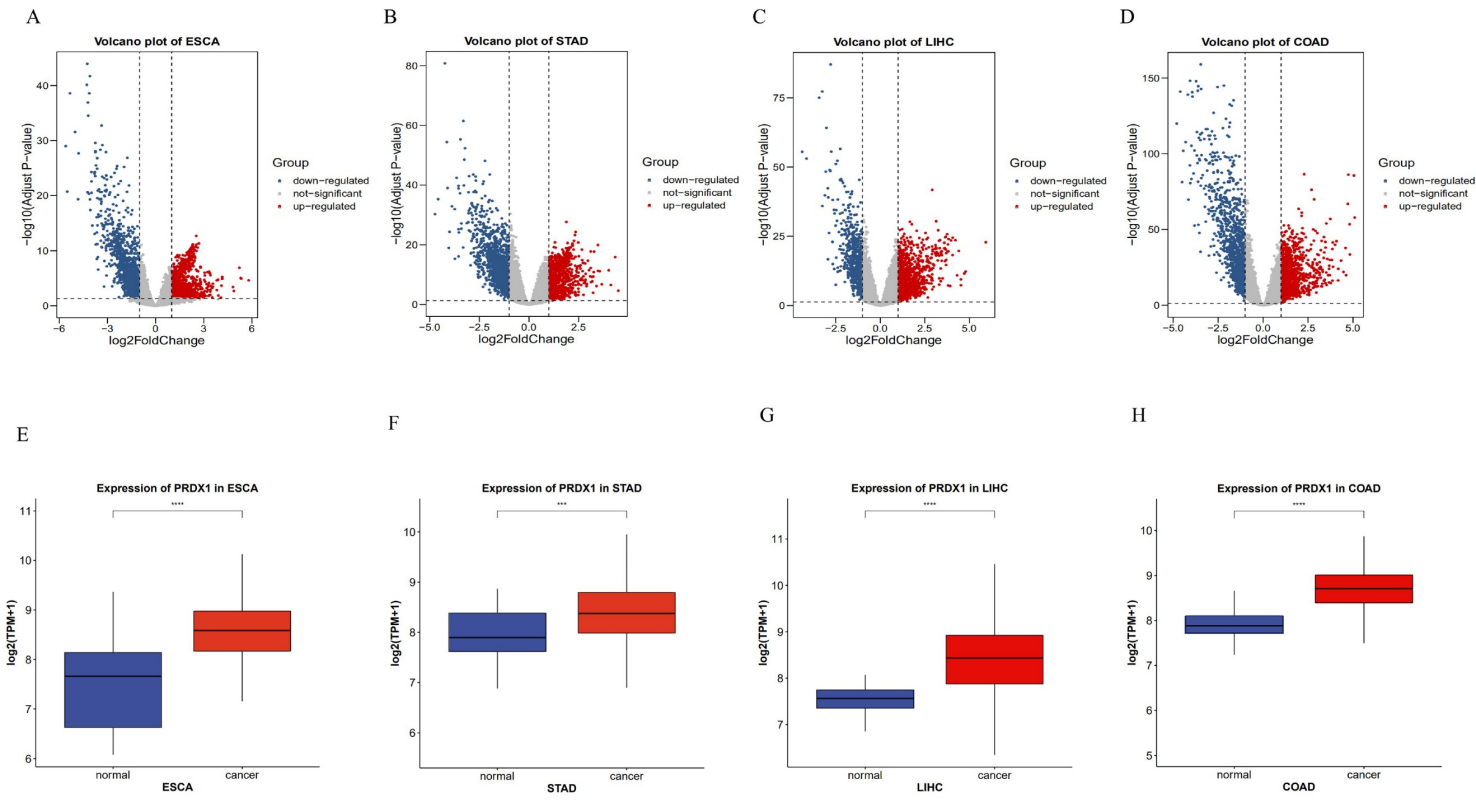
Identification of DEGs of PRDX1 in gastrointestinal cancers. **(A-D)** Volcano plots of gastrointestinal cancers. **(E-H)** Box-plots of PRDX1 in gastrointestinal cancers. ***P<0.001; ****P<0.0001. DEGs, Differentially Expressed Genes.

The R software package was used to identify DEGs of gastrointestinal cancers. DEGs were visualized using microarray volcano plots of gastrointestinal cancers (**Figure 2A-D**). Box-plots were then employed to analyze the expression of PRDX1 in gastrointestinal cancers. When compared to adjacent tissues, PRDX1 is found to be highly expressed in gastrointestinal cancers, with statistical differences noted in all four types of gastrointestinal cancers. This suggests that PRDX1 influences the onset and progression of gastrointestinal cancers (**Figure 2E-H**).

### Relative PRDX1 mRNA Expression in Gastrointestinal Cancers

**Figure 3:**
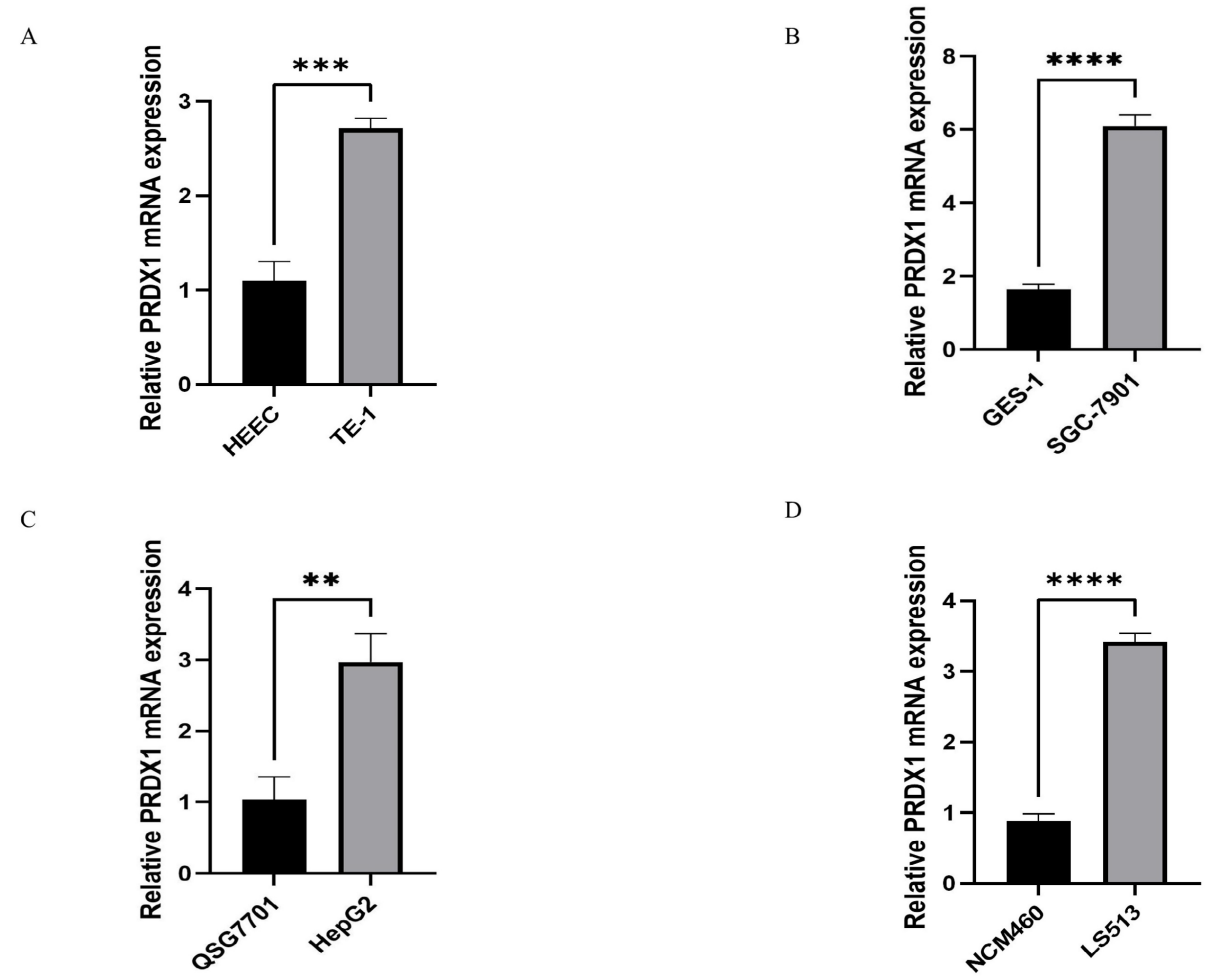
Relative PRDX1 mRNA expression in gastrointestinal cancers. **(A-D)** Relative PRDX1 mRNA expression in gastrointestinal cancers cells. **P<0.01; ***P<0.001; ****P<0.0001.

RT-qPCR technology was used to detect the relative expression of PRDX1 in four types of gastrointestinal cancer cells and corresponding normal cells to validate the results of bioinformatics. The following results were found. The expression of PRDX1 in TE-1 cell is higher than that in HEEC cell, and the difference is statistically significant(p<0.001) (**Figure 3A**); The expression of PRDX1 in SGC-7901 cell is higher than that in GES-1 cell, and the difference is statistically significant(p<0.0001) (**Figure 3B**); The expression of PRDX1 in HepG2 cell is higher than that in QSG7701 cell, and the difference is statistically significant(p<0.01) (**Figure 3C**); The expression of PRDX1 in LS513 cell is higher than that in NCM460 cell, and the difference is statistically significant (p<0.0001) (**Figure 3D**).

### Gene Mutations and Related Genes Analysis of PRDX1 in Gastrointestinal Cancers

The gene mutations of PRDX1 in gastrointestinal cancers were scrutinized, revealing that these mutations predominantly involve amino acid changes, with Missense_ Mutation and Single Nucleotide Polymorphism (SNP) being the main mechanisms (**Table 2**). Following this, related genes analysis of PRDX1 in gastrointestinal cancers was conducted. The analysis indicates that three other types of gastrointestinal cancers exhibit some related genes, but gastric cancer does not (**Table 3**).

### Diagnostic Efficacy of PRDX1 in Gastrointestinal Cancers

**Figure 4:**
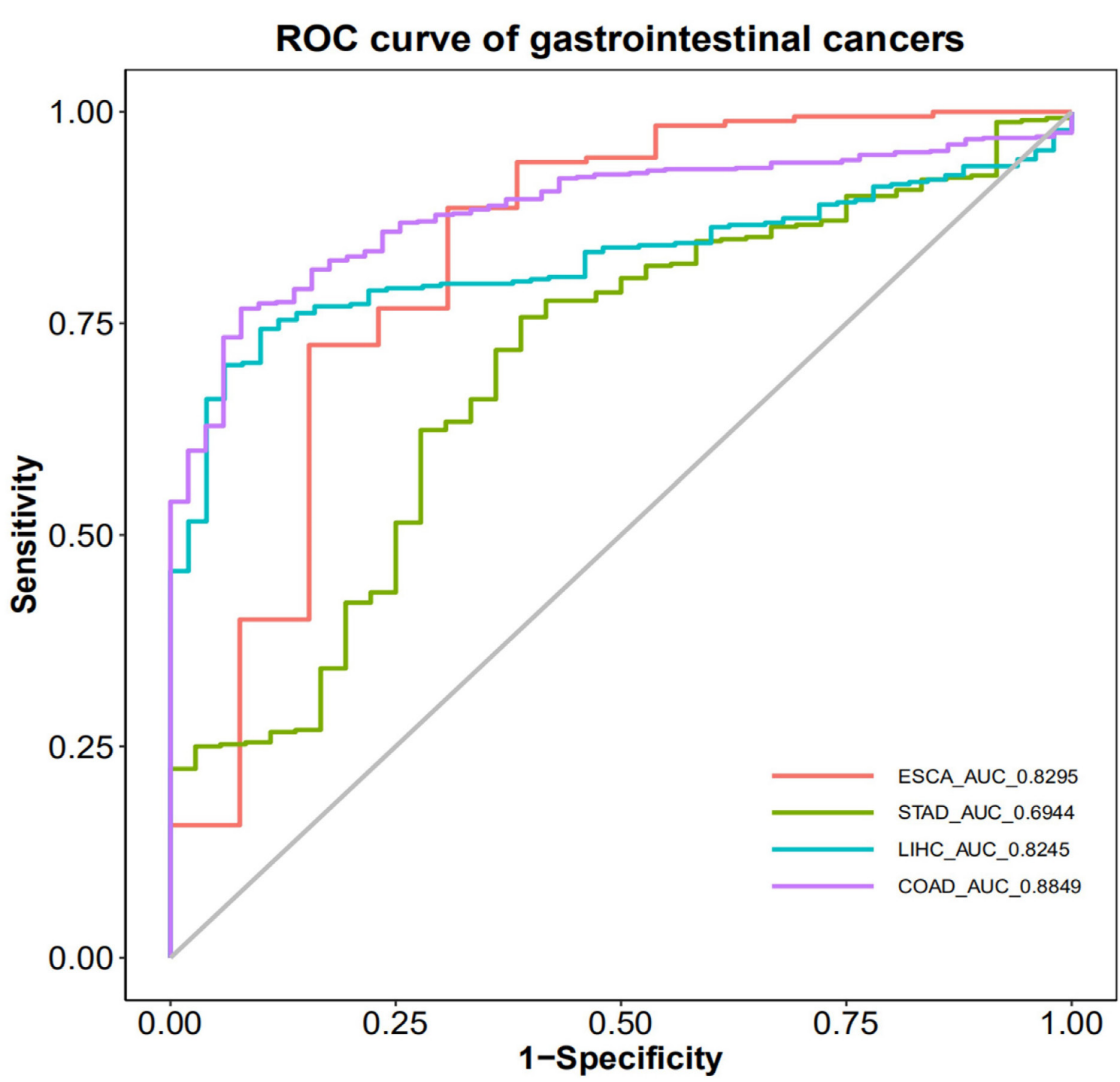
Diagnostic efficacy of PRDX1 in gastrointestinal cancers.

The Receiver Operating Characteristic (ROC) curve was used to illustrate the diagnostic efficacy of PRDX1 in gastrointestinal cancers. The results showed that the Areas Under the Curve (AUC) for esophageal cancer, gastric cancer, liver cancer, and colorectal cancer were 0.8295, 0.6944, 0.8245, and 0.8849, respectively. The cut-off values for these cancers were 0.7866, 0.7982, 0.7899, and 0.8368, respectively. The specificity for these cancers were 0.692, 0.611, 0.900, and 0.922, while the sensitivities were 0.886, 0.757, 0.743, and 0.767, respectively. When the Areas Under the Curve (AUC) is more than 0.7, we believe that this indicator has general diagnostic value for disease detection. When the Areas Under the Curve (AUC) is more than 0.9, we believe that this indicator has great diagnostic value for disease detection. Obviously, PRDX1 has general diagnostic value for esophageal cancer, liver cancer, and colorectal cancer, while its diagnostic value for gastric cancer is relatively low. Therefore, PRDX1 demonstrates the highest diagnostic efficiency for colorectal cancer and the lowest for gastric cancer (**Figure 4**).

### Survival Analysis of PRDX1 in Gastrointestinal Cancers

**Figure 5:**
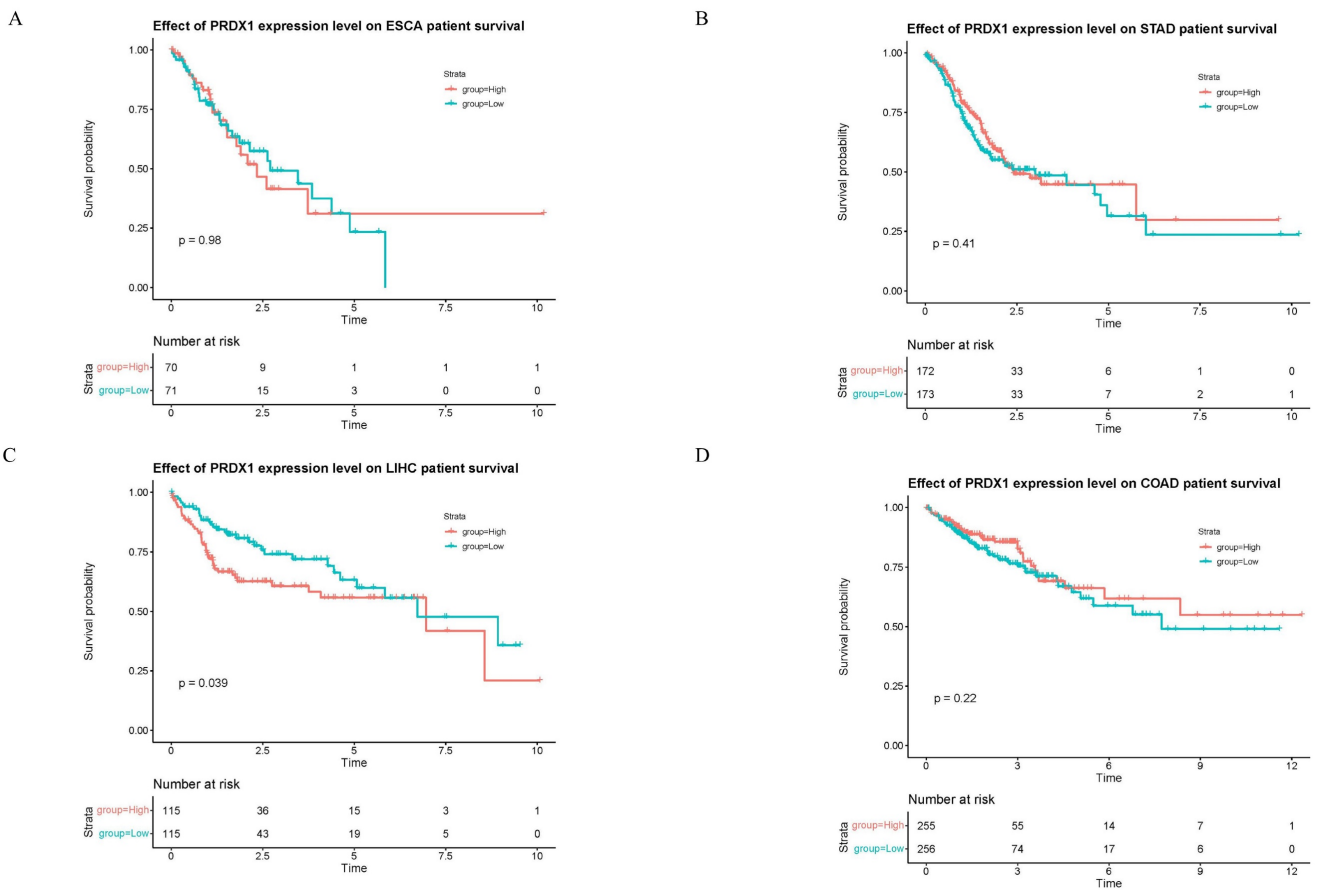
Survival analysis of PRDX1 in gastrointestinal cancers. **(A-D)** Effect of PRDX1 expression level on gastrointestinal cancers patient survival.

The relationship between PRDX1 and survival prognosis of four types of gastrointestinal cancers was investigated (**Figure 5**). Intriguingly, only in liver cancer does the high expression of PRDX1 significantly reduce patient survival time, with the difference deemed statistically significant (p<0.05). No statistical difference was noted among the three other types of gastrointestinal cancers. This suggests that PRDX1 might hold greater significance in liver cancer (**Figure 5C**).

### Origin and Biological Function of PRDX1

**Figure 6:**
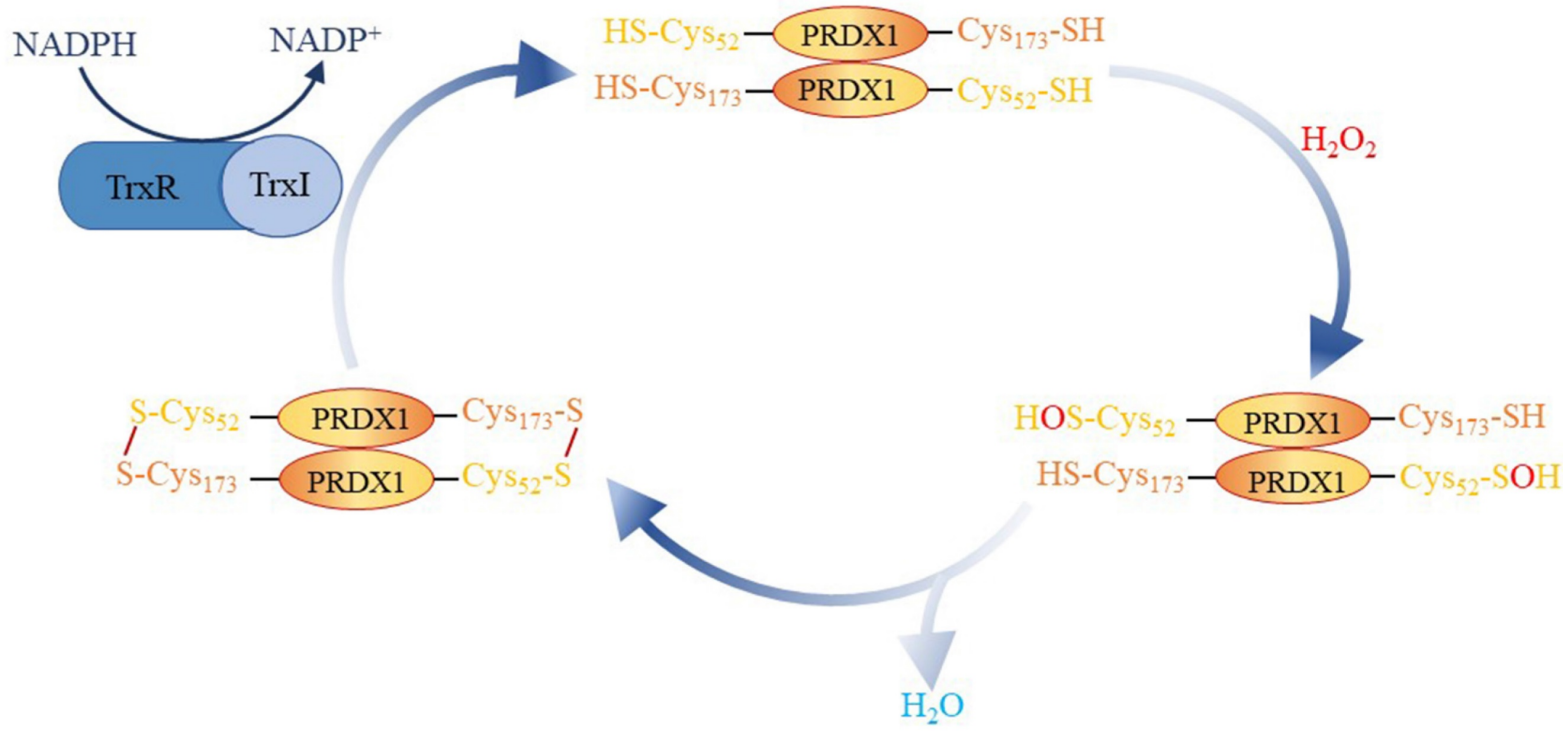
Involvement of PRDX1 in redox cycle mechanism.

In 1988, a protective protein scavenging sulfur free radicals was first discovered in yeast, even though it did not exhibit any known catalytic antioxidant activity akin to catalase, glutathione peroxidase, or superoxide dismutase^24^. Currently, this protective protein is part of the peroxiredoxin (PRDX) protein family. PRDXs, divided into three types based on the number of cysteine residues involved in redox reactions: typical 2-Cys (PRDX1-4), atypical 2-Cys (PRDX5), and 1-Cys (PRDX6)^9^, ^25^, ^26^, have six subtypes, PRDX1-6. PRDX1, an important member of the PRDX family, was originally identified as a human erythrokine, which can boost the activity of natural killer cells^27^. Cys52 in PRDX1 reduces H2O2 to H2O and is oxidized to sulfite. Cys173 reacts with sulfite to form intramolecular disulfide bonds, which are reduced by thioredoxin I (Trx I). Following this, Trx I is reduced by NADPH dependent thioredoxin reductase, creating an oxidation-reduction cycle mechanism (**Figure 6**)^28^. Previous studies have suggested that PRDX or Trx peroxidases are essential endogenous antioxidants that protect cells from oxidative damage by reducing H2O2 and peroxynitrite, as well as by eliminating sulfhydryl radicals^29^-^31^. Nonetheless, the activity of PRDX1 is closely related to the post-translational modification of its protein. For instance, it has been observed that following acetylation at the K197 site of PRDX1, its ability to eliminate Reactive Oxygen Species (ROS) diminishes 32. Furthermore, Mammalian sterile tweenty 1 (Mst1) deactivates PRDX1 by phosphorylating the Thr90 and Thr183 sites of PRDX1, leading to the accumulation of hydrogen peroxide in the cell 33. Chang et al. discovered that cyclin D kinase Cdc2 can also phosphorylate the Thr90 site of PRDX1 and inactivate PRDX1's peroxidase activity^34^. Pin1 binds to PRDX1 through its phospho-Thr90-Pro91 motif. When the Thr90 mutation of PRDX1 occurs, this binding no longer exists^35^. Assays revealed that PRDX1 can be glutathioneized at Cys 52, 83, and 173^36^. This suggests that the post-translational modification of PRDX1 can influence its biological function. Post-translational modification and associated sites of PRDX1 are detailed in **Table 4**.

### Expression and Role of PRDX1 in Esophageal Cancer

Esophageal cancer, a malignancy localized in the esophageal epithelium, constitutes 2% of all malignant tumors. This carcinoma chiefly manifests as either esophageal squamous cell carcinoma (ESCC) or esophageal adenocarcinoma, with ESCC being the predominant type^37^. Several studies report an overexpression of PRDX1 in ESCC cells compared to adjacent non-cancerous tissues^38^-^41^. Enhanced PRDX1 expression is implicated in the onset and advancement of ESCC, while diminished PRDX1 expression appears to curb tumorigenesis 42. These observations suggest a possible oncogenic role of PRDX1 in ESCC. Wu et al. 43 reported that miR-375, by directly targeting PRDX1 mRNA, reduces PRDX1 expression levels, thereby inhibiting the proliferation and migration of ESCC cells. A study by Chen et al. 42 identified PRDX1 as a regulatory factor influencing the formation of primary cilia in ESCC cells. PRDX1, by modulating the HEF1/Aurora A/HDAC6 signaling pathway, fosters the loss of primary cilia, contributing to ESCC tumorigenesis and tumor growth. Inhibiting PRDX1, in this context, exerts anti-tumor effects. Song et al. 44 demonstrated high PRDX1 expression in ESCC, revealing that PRDX1 silencing curtails ESCC proliferation and enhances cell apoptosis. The underlying mechanism potentially involves the obstruction of the PI3K/AKT signaling pathway. However, histone deacetylase inhibitor FK228 is known to activate Prdx1, partly via the regulation of promoter histone acetylation, thereby inducing apoptosis and repressing tumor growth 45. Interestingly, another study indicated a low PRDX1 expression in ESCC, exhibiting a significant negative correlation with lymph node metastasis and TNM staging. In contrast to patients with elevated Prdx1 expression, those exhibiting low Prdx1 expression presented shorter overall survival durations. This suggests a potential tumor-suppressive role for PRDX1, with its downregulation contributing to the progression of esophageal squamous cell carcinoma and serving as a significant prognostic marker 46. The exact role of PRDX1 in esophageal cancer, therefore, remains ambiguous, warranting further investigation.

### Expression and Role of PRDX1 in Gastric Cancer

Gastric cancer (GC), ranking as one of the most prevalent malignancies globally, stands as the fourth primary cause of cancer-related fatalities 47. Numerous studies have indicated that PRDX1 mRNA and protein expression levels in GC tissue exceed those in surrounding non-cancerous tissues. High PRDX1 expression positively correlates with lymph node invasion and unfavorable prognosis, making PRDX1 a potential therapeutic and prognostic target for gastric cancer patients 48-50. Wei et al. identified that Prdx1 regulates the invasion and metastasis of GC cell lines by inhibiting E-Ca expression 48. Zhang et al. 51 discovered that in GC, while PRDX1 is overexpressed, miR-596 levels are reduced. Importantly, miR-596 can directly target PRDX1 mRNA to downregulate PRDX1 expression levels, exerting an inhibitory effect on GC cells. Dehydroharmine hydrochloride (HH), an alkaloid compound exhibiting anti-tumor activity, was found by Tan et al. 52 to significantly decrease the invasion and migration ability of GC cells following HH treatment. This decrease correlated with the reduction of MMP-2 and HIF-1 levels, as well as with the expression of PRDX1. These findings suggest that elevated PRDX1 expression in gastric cancer could serve as a reference indicator for poor prognosis.

### Expression and Role of PRDX1 in Liver Cancer

Liver cancer continues to pose a substantial global health threat, with projections estimating over 1 million cases by 2025 53. The most prevalent radical interventions encompass surgical resection and liver transplantation. Yet, most hepatocellular carcinoma (HCC) patients are often diagnosed at an advanced stage, rendering them unsuitable for surgical interventions. Prognosis for patients undergoing chemotherapy and radiation therapy typically remains poor. Thus, exploring the molecular mechanism of HCC pathogenesis, identifying specific biomarkers, and targeting liver cancer cells provide feasible diagnostic and therapeutic strategies 54-56. Various studies reveal high PRDX1 expression in liver cancer, indicating an inverse correlation with the prognosis of HCC patients. Consequently, PRDX1 may serve as a potential biomarker for HCC detection and prognosis, and its inhibitors could be candidate drugs for liver cancer treatment 57-61. Sun et al. 13 observed that PRDX1 knockout activated cleaved caspase-3, caspase-9, Bax protein, and PARP-1, leading to a reduction in Bcl-2 expression and subsequent induction of apoptosis. This also triggered the expression of dynein related protein1 (Drp1), fission1 (Fis1), and dynein2 (Dyn2), promoting mitochondrial fission and inhibiting the proliferation of hepatoma cells. Lou et al. 62 found that PRDX1 silencing elevated the accumulation of ferrous ions and lipid peroxidation in HepG2 cells, thus fostering iron-induced apoptosis in liver cancer. Patricia et al. 63 determined that PRDX1 silencing led to the down-regulation of AFP, osteopontin, and β-catenin transcripts, while γ-E-cadherin and proapoptotic proteins (Bax, caspase-3) transcripts were upregulated. Further, the activity of glutamyl transpeptidase decreased, and alkaline phosphatase and Casp3 activity increased, collectively inhibiting cell proliferation and growth. Kang et al. 64 discovered that an extract of Auricularia auricula, by inhibiting PRDX1, diminished the levels of total glutathione (GSSG/GSH) and superoxide dismutase (SOD), thereby inactivating antioxidant enzymes, impeding cells from processing reactive oxygen species, and promoting apoptosis of liver cancer cells. Consequently, high PRDX1 expression in liver cancer could serve as a diagnostic marker for liver cancer and is linked to poor prognosis, corroborating the findings of bioinformatics.

### Expression and Role of PRDX1 in Colorectal Cancer

Colorectal cancer stands as one of the common gastrointestinal cancers and is the second leading cause of cancer mortality 65. Recent years have witnessed a global increase in the incidence of colorectal cancer, attributable to changes in lifestyle and dietary patterns, with an emerging trend towards younger age at onset 66. Tumor metastasis accounts for a significant cause of mortality in colorectal cancer patients. Therefore, studying the pathogenesis and metastasis mechanism of colorectal cancer, discovering highly specific and sensitive molecular markers, provides significant new targets for colorectal cancer treatment and prognosis assessment, which is crucial for enhancing the prognosis of colorectal cancer 67. Several studies have identified a positive correlation between PRDX1 expression and tumor grade, lymph node metastasis, and vascular density 68, 69. This variability in the ability to invade and migrate tumor cells across different CRC cell lines suggests that PRDX1 might predict prognosis and serve as a potential therapeutic target, regulating tumor metastasis and angiogenesis in colorectal cancer. It could also serve as a biomarker for early detection of cancer progression and identification of potential pathogenesis 70, 71. Xu et al. 72 proposed that the side effects of Celastrol might be mitigated through structural modification, and PRDX1 inhibition appears promising for treating colorectal cancer. Nonetheless, Tai et al. 73 reported that Gynostemma pentaphyllum saponins (GpS) hinder the development of colorectal cancer by upregulating PRDX1 and PRDX2, inhibiting the Ras, RAF/MEK/ERK/STAT, PI3K/AKT/mTOR signaling pathways, and regulating the JNK/p38 MAPK signaling pathway. These findings indicate potential controversies concerning the expression and role of PRDX1 in colorectal cancer.

### Expression and Role of PRDX1 in Other Gastrointestinal Cancers

Some studies indicate a close relationship between PRDX1 and other gastrointestinal cancers such as pancreatic cancer 74, cholangiocarcinoma 75, and oral cancer 76. In pancreatic cancer, there is a significant elevation in PRDX1 expression levels, making it a viable therapeutic target. Park et al. found that naringenin could diminish PRDX1 expression in pancreatic cancer cells, escalate ROS levels in cancer cells, and subsequently upregulate apoptosis signal-regulated kinase 1 (ASK1) to induce apoptosis 77. In cholangiocarcinoma, PRDX1 expression also significantly increases, influencing the occurrence and development of cholangiocarcinoma by regulating SNAT1 expression^75^. In oral cancer, PRDX1 is highly expressed and is significantly correlated with tumor staging, lymph node metastasis, and pathological grading, making it a promising biomarker for predicting lymph node metastasis and prognosis in oral cancer patients^76^. However, these gastrointestinal cancers have a relatively low incidence rate. Moreover, existing studies on PRDX1 in these cancers are limited, warranting further research for more conclusive insights.

## Discussion

Gastrointestinal cancers, encompassing esophageal cancer, gastric cancer, liver cancer, and colorectal cancer, constitute a primary category of malignancies posing significant threats to human health due to their high incidence and mortality rates. Unraveling the molecular mechanisms that underpin the diagnosis and treatment of gastrointestinal cancers is imperative for facilitating early detection and forecasting prognostic outcomes. As a vital component of the PRDXs family, the peroxidase activity of PRDX1 plays a key role in maintaining the equilibrium of intracellular reactive oxygen species (ROS), thereby influencing the onset, progression, and prognosis of cancer 78-80.

This investigation primarily focuses on reporting the expression and mechanism of PRDX1 in gastrointestinal cancers via bioinformatics methods and review of existing literature, with the goal of offering guidance for the diagnosis and prognosis of gastrointestinal cancers. Initial bioinformatics analysis indicates that PRDX1 expression in gastrointestinal cancers exceeds the median level across pan-cancer. Furthermore, the expression of PRDX1 in the majority of cancers is typically higher than in the corresponding adjacent tissues, with this trend being especially pronounced in gastrointestinal cancers. This forms the groundwork for studying the diagnosis and prognosis of PRDX1 in gastrointestinal cancers, aligning with the research findings of Gao et al 81. Subsequent utilization of the TCGA database enabled the creation of volcanic maps for gastrointestinal cancers to highlight differentially expressed genes (DEGs). To further elucidate the expression of PRDX1 in gastrointestinal cancers, box-plots were generated to display PRDX1 expression in four types of gastrointestinal cancers and their adjacent tissues. Results indicated high PRDX1 expression in all four types of gastrointestinal cancers, all of which were statistically significant. And we used RT-qPCR technology to validate the cells of gastrointestinal cancers, and the results were consistent with the box-plot. However, literature review disclosed some discordance regarding the expression level of PRDX1 in esophageal cancer [38, 41, 45, 46]and colorectal cancer (68, 71, 73). The heightened PRDX1 expression in gastric cancer 48, 51 and liver cancer 57, 60 is consistent with the box-plot results.

In effort to delve deeper into the molecular mechanism of PRDX1 in gastrointestinal cancers, a statistical analysis of gene mutations and related genes of PRDX1 in gastrointestinal cancers was conducted. These mutations were characterized primarily by amino acid changes, with Missense_Mutation and SNP being the predominant forms. Additionally, the analysis suggested that three other types of gastrointestinal cancers exhibit related genes, with the exception of gastric cancer. This implies that PRDX1 mutations may be significant in the onset and progression of gastrointestinal cancers. The related genes of PRDX1 set the stage for future exploration of specific PRDX1 molecules in gastrointestinal cancers.

ROC curves were used to demonstrate the diagnostic efficacy of PRDX1 in gastrointestinal cancers. PRDX1 demonstrated the highest diagnostic efficiency for colorectal cancer and the lowest for gastric cancer. No current literature provides clear evidence regarding the diagnostic efficacy of PRDX1 in gastrointestinal cancers.

The prognostic role of PRDX1 in gastrointestinal cancers was examined by presenting the survival curve of PRDX1 in gastrointestinal cancers through the TCGA database. Interestingly, high PRDX1 expression only decreased the survival time in liver cancer patients. No statistical difference was observed among the three other types of gastrointestinal cancers. Literature review also indicated that high PRDX1 expression promotes the proliferation, invasion, and metastasis of liver cancer cells, thereby fostering the onset and progression of liver cancer 57, 60, 82. These findings align with the results of the survival analysis.

However, our study still has some limitations. In this study, we mainly summarized the role of PRDX1 in gastrointestinal cancers by bioinformatics and previous study. In general, our exploration of PRDX1 in gastrointestinal cancers is meaningful. It is a pity that we did not conduct extensive molecular and cellular experiments to confirm the role of PRDX1 in gastrointestinal cancers apart from RT-qPCR technology. Moreover, we did not rely on real patients of gastrointestinal cancers to improve our study. In the future, we will validate and refine our study from these aspects.

In summary, this study provides evidence that PRDX1 is highly expressed in gastrointestinal cancers and is associated with the diagnosis and prognosis of these malignancies, thus offering potential guidance in the clinical management of gastrointestinal cancers.

## Figures and Tables

**Table 1:** Clinical parameters of patients.

	ESCA	STAD	LIHC	COAD
N	141	345	230	511
Age (%)				
≤60	70(49.6)	112(32.5)	121(52.6)	144(28.2)
>60	71(50.4)	233(67.5)	109(47.4)	367(71.8)
Gender (%)				
Female	21(14.9)	125(36.2)	73(31.7)	236(46.2)
Male	120(85.1)	220(63.8)	157(68.3)	275(53.8)
T (%)				
T1	24(17.0)	15(4.3)	113(49.1)	17(3.3)
T2	39(27.7)	71(20.6)	51(22.2)	90(17.6)
T3	74(52.5)	162(47.0)	56(24.3)	353(69.1)
T4	4(2.8)	97(28.1)	10(4.3)	51(10.0)
N (%)				
N0	67(47.5)	109(31.6)	226(98.3)	301(58.9)
N1	60(42.6)	92(26.7)	4(1.7)	120(23.5)
N2	9(6.4)	71(20.6)	0(0)	90(17.6)
N3	5(3.5)	73(21.2)	0(0)	0(0)
M (%)				
M0	132(93.6)	322(93.3)	227(98.7)	429(84.0)
M1	9(6.4)	23(6.7)	3(1.3)	82(16.0)
Stage (%)				
Stage I	13(9.2)	45(13.0)	111(48.3)	94(18.4)
Stage II	72(51.1)	112(32.5)	50(21.7)	197(38.6)
Stage III	47(33.3)	153(44.3)	65(28.3)	138(27.0)
Stage IV	9(6.4)	35(10.1)	4(1.7)	82(16.0)

**Table 2:** Gene mutations of PRDX1 in gastrointestinal cancers.

Cancer Type	Sample ID	Protein Change	Mutation Type	Variant Type
LIHC	TCGA-CC-A7IE-01	TESK2-PRDX1 Fusion	Fusion	NA
LIHC	TCGA-DD-A73G-01	TESK2-PRDX1 Fusion	Fusion	NA
STAD	TCGA-VQ-A8E0-01	TESK2-PRDX1 Fusion	Fusion	NA
COAD	TCGA-EI-6885-01	GOLIM4-PRDX1 Fusion	Fusion	NA
LIHC	TCGA-DD-A3A3-01	F195L	Missense_Mutation	SNP
ESCA	TCGA-JY-A6FE-01	S191R	Missense_Mutation	SNP
ESCA	TCGA-IG-A3QL-01	G129D	Missense_Mutation	SNP
STAD	TCGA-BR-8680-01	E193*	Nonsense_Mutation	SNP
COAD	TCGA-AG-A002-01	E65*	Nonsense_Mutation	SNP
COAD	TCGA-A6-5661-01	A63T	Missense_Mutation	SNP
COAD	TCGA-DM-A1HB-01	Y194H	Missense_Mutation	SNP

**Table 3:** Related genes analysis of PRDX1 in gastrointestinal cancers

Cancer Type	Related Gene	Correlation	Coefficient
ESCA	EPHX1	Postive	0.81
ESCA	GSTM3	Postive	0.77
ESCA	AKR1C1	Postive	0.76
ESCA	MICAL1	Neagive	0.36
ESCA	SH3KBP1	Neagive	0.34
ESCA	CLMN	Neagive	0.33
COAD	PSMB2	Postive	0.65
COAD	EBNA1BP2	Postive	0.61
COAD	HSPB11	Postive	0.6
COAD	SHC2	Neagive	0.35
COAD	GLTSCR2	Neagive	0.34
COAD	EEF2	Neagive	0.33
LIHC	EIF2B3	Postive	0.7
LIHC	PSMA5	Postive	0.68
LIHC	EBNA1BP2	Postive	0.64
LIHC	COL18A1	Neagive	0.33
LIHC	SERPINA5	Neagive	0.33
LIHC	TMPRSS6	Neagive	0.32
STAD	N/A	N/A	N/A

**Table 4:** Post-translational modification of PRDX1 in point mutation.

			Records, n	
Mutation	Flanking sequence	Modification	LTP	HTP
K7	MSSGNAkIGHPAPN	Acetylation	0	4
K7	MSSGNAkIGHPAPN	Ubiquitylation	0	6
K7	MSSGNAkIGHPAPN	Sumoylation	0	1
K7	MSSGNAkIGHPAPN	Succinylation	0	1
K16	GHPAPNFkAtAVMPD	Acetylation	0	13
K16	GHPAPNFkAtAVMPD	Ubiquitylation	0	8
K16	GHPAPNFkAtAVMPD	Sumoylation	0	1
K16	GHPAPNFkAtAVMPD	Methylation	0	1
K16	GHPAPNFkAtAVMPD	Succinylation	0	1
T18	PAPNFkAtAVMPDGQ	Phosphorylation	0	1
K27	VMPDGQFkDIsLsDy	Acetylation	0	15
K27	VMPDGQFkDIsLsDy	Ubiquitylation	0	16
K27	VMPDGQFkDIsLsDy	Methylation	0	1
K27	VMPDGQFkDIsLsDy	Succinylation	0	1
S30	DGQFkDIsLsDykGk	Phosphorylation	0	6
S32	QFkDIsLsDykGkYV	Phosphorylation	1	16
Y34	kDIsLsDykGkYVVF	Phosphorylation	0	8
K35	DIsLsDykGkYVVFF	Acetylation	0	79
K35	DIsLsDykGkYVVFF	Ubiquitylation	0	38
K35	DIsLsDykGkYVVFF	Succinylation	0	1
K37	sLsDykGkYVVFFFY	Acetylation	0	1
K37	sLsDykGkYVVFFFY	Ubiquitylation	0	7
T90	CHLAWVNtPkkQGGL	Phosphorylation	3	2
K92	LAWVNtPkkQGGLGP	Acetylation	0	2
K92	LAWVNtPkkQGGLGP	Succinylation	0	1
K93	AWVNtPkkQGGLGPM	Acetylation	0	1
K93	AWVNtPkkQGGLGPM	Ubiquitylation	0	7
K93	AWVNtPkkQGGLGPM	Sumoylation	0	1
K109	IPLVsDPkRtIAQDy	Acetylation	0	67
K109	IPLVsDPkRtIAQDy	Ubiquitylation	0	14
K109	IPLVsDPkRtIAQDy	Succinylation	0	1
K120	AQDyGVLkADEGIsF	Ubiquitylation	0	15
K120	AQDyGVLkADEGIsF	Succinylation	0	1
K136	GLFIIDDkGILRQIT	Acetylation	0	5
K136	GLFIIDDkGILRQIT	Ubiquitylation	0	18
K168	QAFQFtDkHGEVCPA	Acetylation	0	7
K168	QAFQFtDkHGEVCPA	Ubiquitylation	0	7
K178	EVCPAGWkPGsDtIk	Acetylation	0	2
K178	EVCPAGWkPGsDtIk	Ubiquitylation	0	18
K185	kPGsDtIkPDVQkSk	Acetylation	0	2
K185	kPGsDtIkPDVQkSk	Ubiquitylation	0	8
K185	kPGsDtIkPDVQkSk	Sumoylation	0	1
K192	kPDVQkSkEyFskQK	Acetylation	0	3
K192	kPDVQkSkEyFskQK	Ubiquitylation	0	9
K192	kPDVQkSkEyFskQK	Methylation	0	4

LTP (Low Throughput Papers), the number of records in which this modification site was determined using methods other than discovery mass spectrometry; HTP (High Throughput Papers), the number of records in which this modification site was assigned using only proteomic discovery mass spectrometry.

## Abbreviations

AUC: area under the curve
PRDX1: peroxiredoxin 1
TCGA: the cancer genome atlas
GTEx: genotype-tissue expression
ACC: adrenocortical carcinoma
BLCA: bladder urothelial carcinoma
BRCA: breast invasive carcinoma
CESC: cervical squamous cell carcinoma
CHOL: cholangiocarcinoma
COAD: colorectal adenocarcinoma
DLBC: Lymphiod neoplasm diffuse large B-cell lymphoma
ESCA: esophageal carcinoma
GBM: glioblastoma multiforme
HNSC: head and Neck squamous cell carcinoma
KICH: kidney chromophobe
KIRC: kidney renal clear cell carcinoma
KIRP: kidney renal papillary cell carcinoma
LGG: brain lower grade glioma
OV: ovarian serous cystadenocarcinoma
MESO: mesothelioma
LIHC: liver hepatocellular carcinoma
LUAD: lung adenocarcinoma
LUSC: lung squamous cell carcinoma
PAAD: pancreatic adenocarcinoma
PRAD: prostate adenocarcinoma
PCPG: pheochromocytoma and Paraganglioma
READ: Rectum adenocarcinoma
SARC: sarcoma
SKCM: skin cutaneous melanoma
LAML: acute myeloid leukemia
TGCT: testis germ cell tumors
THCA: thyroid carcinoma
THYM: thymoma
STAD: stomach adenocarcinomna
UCEC: uterine corpus endometrial carcinoma
UCS: uterine carcinosarcoma
UVM: uveal melanoma
